# Colistin Susceptibility in Companion Animal-Derived *Escherichia coli, Klebsiella* spp., and *Enterobacter* spp. in Japan: Frequent Isolation of Colistin-Resistant *Enterobacter cloacae* Complex

**DOI:** 10.3389/fcimb.2022.946841

**Published:** 2022-07-06

**Authors:** Toyotaka Sato, Kazuki Harada, Masaru Usui, Shin-ichi Yokota, Motohiro Horiuchi

**Affiliations:** ^1^ Laboratory of Veterinary Hygiene, School/Faculty of Veterinary Medicine, Hokkaido University, Sapporo, Japan; ^2^ Graduate School of Infectious Diseases, Hokkaido University, Sapporo, Japan; ^3^ Department of Microbiology, Sapporo Medical University School of Medicine, Sapporo, Japan; ^4^ Department of Veterinary Internal Medicine, Tottori University, Tottori, Japan; ^5^ Laboratory of Food Microbiology and Food Safety, Department of Health and Environmental Sciences, School of Veterinary Medicine, Rakuno Gakuen University, Ebetsu, Japan

**Keywords:** bacteria, Enterobacterales, *Enterobacter* spp, antimicrobial resistance, colistin

## Abstract

Transmission of colistin-resistant *Enterobacterales* from companion animals to humans poses a clinical risk as colistin is a last-line antimicrobial agent for treatment of multidrug-resistant Gram-negative bacteria including *Enterobacterales*. In this study, we investigated the colistin susceptibility of 285 *Enterobacterales* (including 140 *Escherichia coli*, 86 *Klebsiella* spp., and 59 *Enterobacter* spp.) isolated from companion animals in Japan. We further characterized colistin-resistant isolates by multilocus sequence typing (MLST), phylogenetic analysis of *hsp60* sequences, and population analysis profiling, to evaluate the potential clinical risk of companion animal-derived colistin-resistant *Enterobacterales* to humans in line with the One Health approach. All *E. coli* isolates were susceptible to colistin, and only one *Klebsiella* spp. isolate (1.2%, 1/86 isolates) was colistin resistant. *Enterobacter* spp. isolates were frequently colistin resistant (20.3%, 12/59 isolates). In colistin-resistant *Enterobacter* spp., all except one isolate exhibited colistin heteroresistance by population analysis profiling. These colistin-heteroresistant isolates belonged to clusters I, II, IV, VIII, and XII based on *hsp60* phylogeny. MLST analysis revealed that 12 colistin-resistant *Enterobacter* spp. belonged to the *Enterobacter cloacae* complex; five *Enterobacter kobei* (four ST591 and one ST1577), three *Enterobacter asburiae* (one ST562 and two ST1578), two *Enterobacter roggenkampii* (ST606 and ST1576), and *Enterobacter hormaechei* (ST1579) and *E. cloacae* (ST765) (each one strain). Forty-two percent of the colistin-resistant *E. cloacae* complex isolates (predominantly ST562 and ST591) belonged to lineages with human clinical isolates. Four *E. kobei* ST591 isolates were resistant to third-generation cephalosporines, aminoglycosides, and fluroquinolones but remained susceptible to carbapenems. In conclusion, our study is the first to our knowledge to report the frequent isolation of the colistin-resistant *E. cloacae* complex from companion animals. Furthermore, a subset of isolates belonged to human-associated lineages with resistance to multiple classes of antibiotics. These data warrant monitoring carriage of the colistin-resistant *E. cloacae* complex in companion animals as part of a domestic infection control procedure in line with the One Health approach.

## Introduction

Colistin is a last-line antimicrobial agent used for treatment of multidrug-resistant Gram-negative bacteria, including *Enterobacterales* (Falagas and Kasiakou, 2005). Spread of colistin resistance therefore poses a major therapeutic problem for the treatment of human infections. The plasmid-mediated colistin resistance gene, *mcr*, has spread worldwide (Hussein et al., 2021). *mcr* encodes a phosphoethanolamine transferase and reduces colistin susceptibility by modifying the lipid A component of lipopolysaccharide with phosphoethanolamine (Hussein et al., 2021). The *mcr* variants from *mcr-1* to *mcr-10* have been known, whereas few countries have reported *mcr-9* and *mcr-10* (Wang et al., 2020). Recently, we identified *mcr-9* and *mcr-10* in *Enterobacter asburiae* and *Enterobacter roggenkampii*, respectively, from companion animals in Japan (Sato et al., 2021a; Sato et al., 2021b). These genes were encoded by plasmids with high sequence similarity to those from human clinical isolates (Sato et al., 2021a; Sato et al., 2021b). These observations suggested that horizontal transfer of the plasmid encoding *mcr-9* and *mcr-10* occurs between isolates from humans and companion animals, which may pose a major risk to human health. However, the overall risk of transmission of colistin-resistant *Enterobacterales* from companion animals to humans was unclear, partly because of an incomplete characterization of carriage of these organisms in companion animals.

In this study, we investigated the colistin susceptibility of *Enterobacterales* isolates derived from companion animals in Japan and characterized colistin-resistant isolates using molecular epidemiological analysis.

## Materials and Methods

### Bacterial Isolates

Our bacterial collection encompassed 285 *Enterobacterales* [140 *Escherichia coli*, 86 *Klebsiella* spp. (84 *Klebsiella pneumoniae* and two *Klebsiella quasipneumoniae*), and 59 *Enterobacter* spp. (51 *Enterobacter cloacae* complex and eight *Enterobacter aerogenes*)]. These isolates were derived from clinical specimens of 285 companion animals (211 dogs and 74 cats) from 2003 to 2016 at veterinary clinics throughout Japan (Harada et al., 2012; Harada et al., 2016; Harada et al., 2017). This study was carried out in accordance with the Guidelines for Proper Conduct of Animal Experiments of the Science Council of Japan (Harada et al., 2012; Harada et al., 2016; Harada et al., 2017). Bacterial strains were isolated as part of a routine veterinary examination without invasive operations on the companion animals.

### Antimicrobial Susceptibility

Susceptibility to colistin was determined using the broth microdilution method according to the Clinical Laboratory Standard Institution (CLSI) guidelines (Clinical and Laboratory Standards Institute, 2020). *E. coli* strain ATCC 25922 was used as a reference. Isolates were classified as resistant if their colistin minimum inhibitory concentrations (MIC) was greater than 4 mg/l according to CLSI breakpoint guidelines (Clinical and Laboratory Standards Institute, 2020). To evaluate the “skip-well” phenomenon, bacterial density in each well of the microbroth dilution plate was determined by measuring their optical density at 600nm (OD_600nm_) using a TECAN Infinite M200 PRO microplate reader (Tecan, Kawasaki, Japan). OD_600nm_ values of less than 0.1 were considered as no growth. Antimicrobial susceptibilities of a subset of isolates to cefotaxime, ceftazidime, meropenem, gentamicin, and ciprofloxacin were reported previously (Harada et al., 2017).

### Heat Shock Protein (*hsp60*) Phylogeny

The *hsp60* phylogeny was determined as previously reported (Hoffmann and Roggenkamp, 2003). Nucleotide sequences were aligned using MAFFT (Katoh et al., 2019), and MEGA X (Kumar et al., 2018; Stecher et al., 2020) was used to generate a phylogenetic tree using the Neighbor-Joining method (Saitou and Nei, 1987). The reference strains of each cluster were used as previously reported (Hoffmann and Roggenkamp, 2003).

### Genome Sequencing and Identification of STs and Antimicrobial Resistance Genes

Genomic DNA was isolated using the DNeasy Blood & Tissue Kit (Qiagen, Hilden, Germany). Genomic libraries were prepared by Nextera DNA Flex Library Prep for Illumina sequencing according to the manufacturer’s instructions (Illumina, San Diego, CA). Genome sequencing was performed using the HiSeq 2000 instrument (Illumina). Filtered subreads (in fastq format) were *de novo* assembled with 300-bp paired-end reads after trimming using the CLC Genomic Workbench (Qiagen). The sequence data (in.fasta format) were used to identify *E. cloacae* complex species by KmerFinder 3.2 and sequence types (STs) by multilocus sequence typing (MLST) analysis. Antimicrobial resistance genes were identified using ResFinder from the Center for Genomic Epidemiology (http://www.genomicepidemiology.org).

### Population Analysis Profiling

An evaluation of colistin heteroresistance by population analysis profiling (PAP) was carried out as previously described (Guerin et al., 2016). Briefly, isolates were inoculated into 1 ml of Tryptic soy broth (Becton Dickinson GmbH, Heidelberg, Germany) and incubated at 37°C overnight. One hundred microliters of each serial dilution was plated on Mueller Hinton II (MHII) agar plates (Becton Dickinson GmbH) supplemented with 0, 0.125, 0.5, or 8 mg/l of colistin and cultured for 20 h at 37°C.

After counting the number of colony-forming units (cfu), the frequency of the population displaying colistin resistance was calculated by dividing the number of cfu on colistin-containing MHII agar by the cfu on plain-MHII agar. Colistin heteroresistance was determined using a previously published method based on the following criteria (Andersson et al., 2019). 1) Bacterial growth was confirmed in >4 mg/l of colistin by microbroth dilution, and 2) the colistin concentration exhibiting the highest inhibitory effect on the growth of the resistant subpopulation (with a frequency range from 1 × 10^-5^ to 1 × 10^-1^) was at least eight-fold greater (= 8 mg/l of colistin) than the highest colistin concentration exhibiting no effect on the growth of the colistin-susceptible population (= 0.125 or 0.5 mg/l of colistin) as described in previous studies (Guerin et al., 2016; Juhasz et al., 2017).

## Results

### Colistin Susceptibility in *Enterobacterales* spp. Derived From Companion Animals

**Figure 1:**
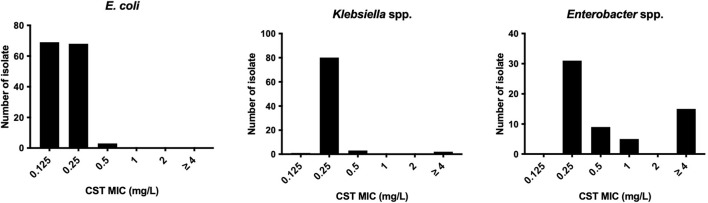
Colistin susceptibility in *E. coli*, *Klebsiella* spp., and *Enterobacter* spp. clinical isolates derived from companion animals.

**Figure 2:**
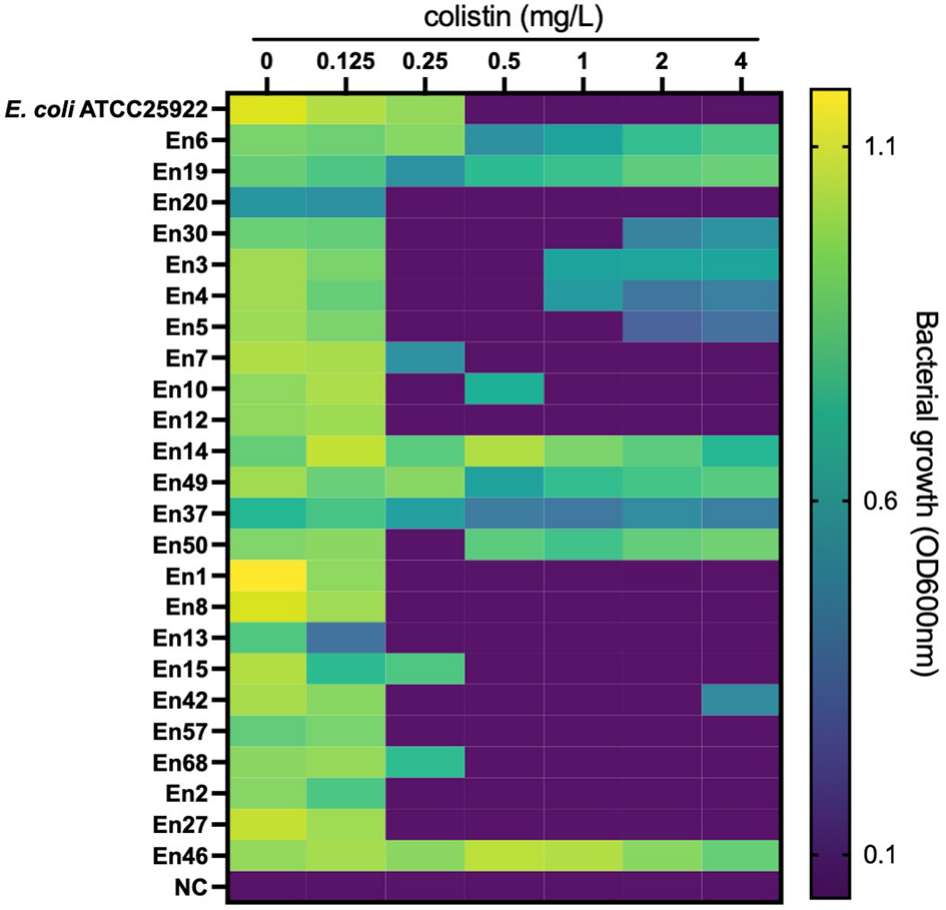
Bacterial growth in colistin-containing medium to evaluate the ‘skip-well’ phenomenon in *Enterobacter* spp. Isolates This method was identical to the broth microdilution method described in the CLSI guidelines. Bacterial growth was determined as turbidity of the medium in cation-adjusted MHII broth at the experimental endpoint (after an 18-h cultivation at 37°C in a 96-well plate). OD_600nm_ was measured every 10 min for 18 h. An OD_600nm_ of <0.1 was defined as no growth.

All *E. coli* isolates tested were susceptible to colistin, with MIC ranging from ≤0.125 to 0.5 mg/l (MIC_50_ and MIC_90_ were 0.125 and 0.25 mg/l, respectively) (**Figure 1**). In *Klebsiella* spp., only one isolate of *K. pneumoniae* (1.2%, 1/86 isolates) was colistin resistant with MICs ranging from ≤0.125 to >4 mg/l (MIC_50_ and MIC_90_ were both 0.25 mg/l). Instead, the colistin MICs of *Enterobacter* spp. ranged from 0.25 to >4 mg/l (MIC_50_ and MIC_90_, 0.25 and >4 mg/l, respectively), and the frequency of colistin resistance was significantly higher (20.3%, 12/59 isolates) than that of *E. coli* and *Klebsiella* spp. (*p* < 0.05). All of the 12 colistin-resistant isolates belonged to the *E. cloacae* complex including *Enterobacter kobei* (8.5%), *E. asburiae* (5.1%), *E. roggenkampii* (3.4%), *Enterobacter hormaechei*, and *E. cloacae* (each 1.7%), and no colistin-resistant *E. aerogenes* was observed. Six colistin-resistant *Enterobacter* isolates (En3, En4, En4, En30, En42, and En50 strains) exhibited a “skip-well” phenotype whereby they grew in the presence of 2 mg/l colistin, but not within the range of 0.25 to 1 mg/l colistin (**Figure 2**).

### Antimicrobial Susceptibility and Detection of Antimicrobial Resistance Genes

According to clinical breakpoints, 50.0% (6 isolates) and 41.6% (n = 5 isolates) of 12 colistin-resistant *E. cloacae* complex isolates exhibited resistance to fluoroquinolones (ciprofloxacin) and third-generation cephalosporins (cefotaxime and ceftazidime), and aminoglycosides (gentamicin), respectively, whereas they remained susceptible to carbapenem (meropenem) (**Table 1**). Furthermore, 33.3% (4/12 isolates, En3, En4, En5, and En14) of the colistin-resistant isolates were co-resistant to ciprofloxacin, third-generation cephalosporins, and gentamicin.

**Table 1:** Characteristics of colistin-resistant *Enterobacter cloacae* complex isolates derived from companion animals.

Strains	*Enterobacter* spp.	Origins	Specimens	Antimicrobial susceptibility (MIC, mg/L)						*hsp60* cluster	MLST	Antimicrobial resistance genes
				CTX	CAZ	MEM	GEN	CIP	CST			
En3	*E. kobei*	Dog	Urine	64^a^	128^a^	<0.125^a^	>128^a^	64^a^	>128^b^	II	ST591^c^	*catA2, sul1, sul2, aac(6′)-Ib3, aac(6′)-IIc, ere(A), qnrB2, aac(6′)-Ib-cr, tet(D), fosA, bla_TEM-1B_, bla_SHV-12_, bla_ACT-9_*
En4	*E. kobei*	Dog	Urine	64^a^	128^a^	<0.125^a^	>128^a^	64^a^	>128^b^	II	ST591^c^	*catA2, sul1, sul2, aac(6′)-Ib3, aac(6′)-IIc, ere(A), qnrB2, aac(6′)-Ib-cr, tet(D), fosA, bla_TEM-1B_, bla_SHV-12_, bla_ACT-9_*
En5	*E. kobei*	Dog	Urine	64^a^	>128^a^	<0.125^a^	>128^a^	64^a^	>128^b^	II	ST591^c^	*catA2, sul1, sul2, aac(6′)-Ib3, aac(6′)-IIc, ere(A), qnrB2, aac(6′)-Ib-cr, tet(D), fosA, bla_TEM-1B_, bla_SHV-12_, bla_ACT-9_*
En14	*E. kobei*	Cat	Kidney	32^a^	128^a^	<0.125^a^	128^a^	128^a^	>128	II	ST591^c^	*tet(D), catA2, bla_TEM-1B_, bla_SHV-12_, bla_ACT-9_, qnrB2, fosA, ere(A), aac(6′)-IIc, sul1, sul2*
En49	*E. kobei*	Dog	Urine	0.25	0.25	<0.125	0.5	<0.125	>128	II	ST1577	*fosA, bla_ACT-9_*
En6	*E. asburiae*	Dog	Prostate	16^a^	32^a^	<0.125^a^	0.5^a^	4^a^	>128	I	ST1578	*aph(3”)-Ib, aph(6)-Id, fosA, bla_ACT-10_*
En19	*E. asburiae*	Cat	Skin	0.125	0.25	<0.125	0.25	<0.125	>128	I	ST562^c^	*bla*_ACT-3_, *qnrE1*
En30	*E. asburiae*	Cat	Nasal cavity	0.5	2	<0.125	>128	16	>128^b^	I	ST1578	*catA2, sul1, tet(D), mph(A), aac(6′)-Ib3, aph(6)-Id, aph(3”)-Ib, aac(3)-IIa, aac(6′)-Ib-cr, qnrB4, fosA, dfrA19, dfrA17, bla_DHA-1_, bla_ACT-10_, bla_TEM-1B_, mcr-9*
En37	*E. roggenkampii*	Dog	Pus	<0.125	0.24	<0.125	0.5	<0.125	>128	IV	ST1576	*bla_MIR-2_, mcr-10*
En50	*E. roggenkampii*	Cat	Urine	0.5	0.5	<0.125	0.5	<0.125	>128^b^	IV	ST606	*fosA, bla_MIR-3_*
En42	*E. hormaechei*	Dog	Urine	0.5	0.5	<0.125	0.5	<0.125	>128^b^	VIII	ST1579	*bla_ACT-7_*
En46	*E. cloacae*	Dog	Pus	0.25	0.5	<0.125	1	<0.125	>128	XII	ST765	*fosA, bla_CMH-3_*

CTX, cefotaxime; CAZ, ceftazidime; MEM, meropenem; CIP, ciprofloxacin; CST, colistin; a, previously determined (Harada et al., 2017); b, “skip-well” phenotype was observed during the colistin susceptibility test; c, identical STs with human clinical isolates (Wang et al., 2017; Zhou et al., 2018).

Using ResFinder, we found that two colistin-resistant *E. cloacae* complex isolates possessed *mcr-9* (*E. asburiae* En30) (Sato et al., 2021a) and *mcr-10* (*E. roggenkampii* En37) (Sato et al., 2021b), as previously reported. Other colistin-resistant *E. cloacae* complex isolates did not possess *mcr* genes. *E. kobei* En3, En4, En5, and En14 possess the extended-spectrum β-lactamase gene *bla*_SHV-12_, in addition to the class A and *ampC* β-lactamase genes *bla*_TEM-1B_ and *bla*_ACT-9_, respectively (**Table 1**). Additional β-lactamase genes (*bla*_ACT-3_, *bla*_ACT-7_, *bla*_ACT-10_, *bla*_DHA-1_, *bla*_MIR-2_, *bla*_MIR-3_, *bla*_CMH-3_) were detected in other colistin-resistant isolates. The quinolone resistance gene *qnrB2* was detected in five colistin-resistant *E. cloacae* complex isolates, and *qnrE1* was also detected in the colistin-resistant strain (En19). The aminoglycoside resistance genes, *aph(3″)-Ib*, *aph(6)-Id*, *aac(3)-IIa*, a*ac(6′)-Ib3*, *aac(6′)-IIc*, and/or *aac(6′)-Ib-cr*, were also detected in colistin-resistant *E. cloacae* complex isolates (**Table 1**).

### *hsp60* Phylogeny and MLST Analysis

**Figure 3:**
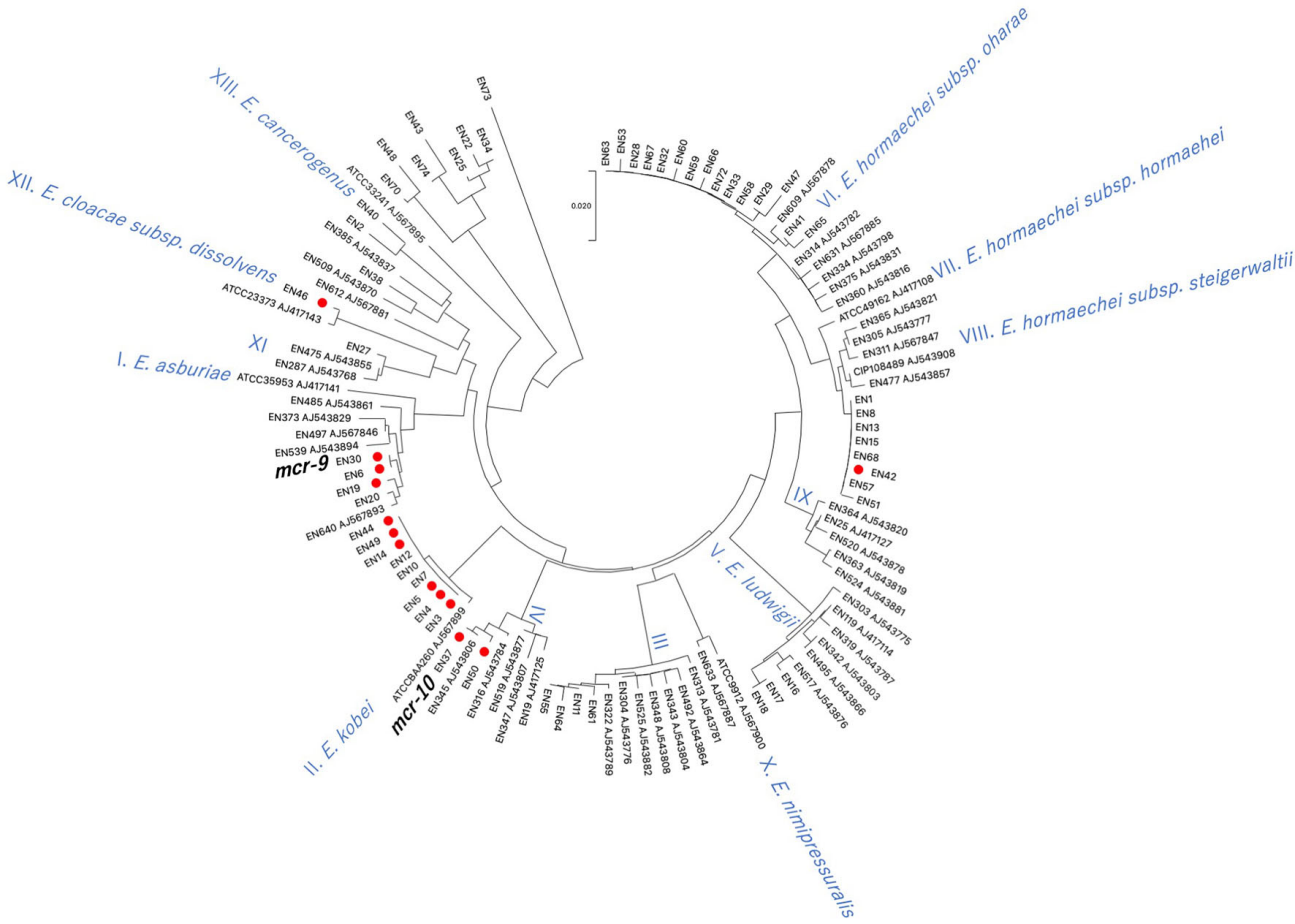
Phylogenetic tree based on *hsp60* sequences in *Enterobacter* spp. isolates derived from companion animals Red circles indicate colistin-resistant isolates. Reference strains are denoted by their respective accession numbers.

Using *hsp60* phylogeny, the *Enterobacter* spp. isolates were found to belong to 10 clusters (I, II, III, IV, V, VI, VIII, XI, XII, XIII) (**Figure 3**). Among them, colistin-resistant isolates belonged to five clusters (I, II, IV, VIII, and XII). Cluster II contained the majority of colistin-resistant isolates (5/12 colistin-resistant isolates) followed by clusters I (three isolates), IV (two isolates), and VIII, and XII (one strain each). Profiling by MLST analysis revealed that four and a colistin-resistant *E. kobei* isolate belonging to cluster II were ST591 and ST1577, respectively (**Table 1**). Colistin-resistant isolates of cluster I contained *E. asburiae* ST562 and ST1578, and those of cluster IV were *E. roggenkampii* ST606 and ST1576. Colistin-resistant isolates from clusters VIII and XII consisted of *E. hormaechei* ST1579 and *E. cloacae* ST765, respectively.

### PAP of the Colistin-Resistant Population

**Figure 4:**
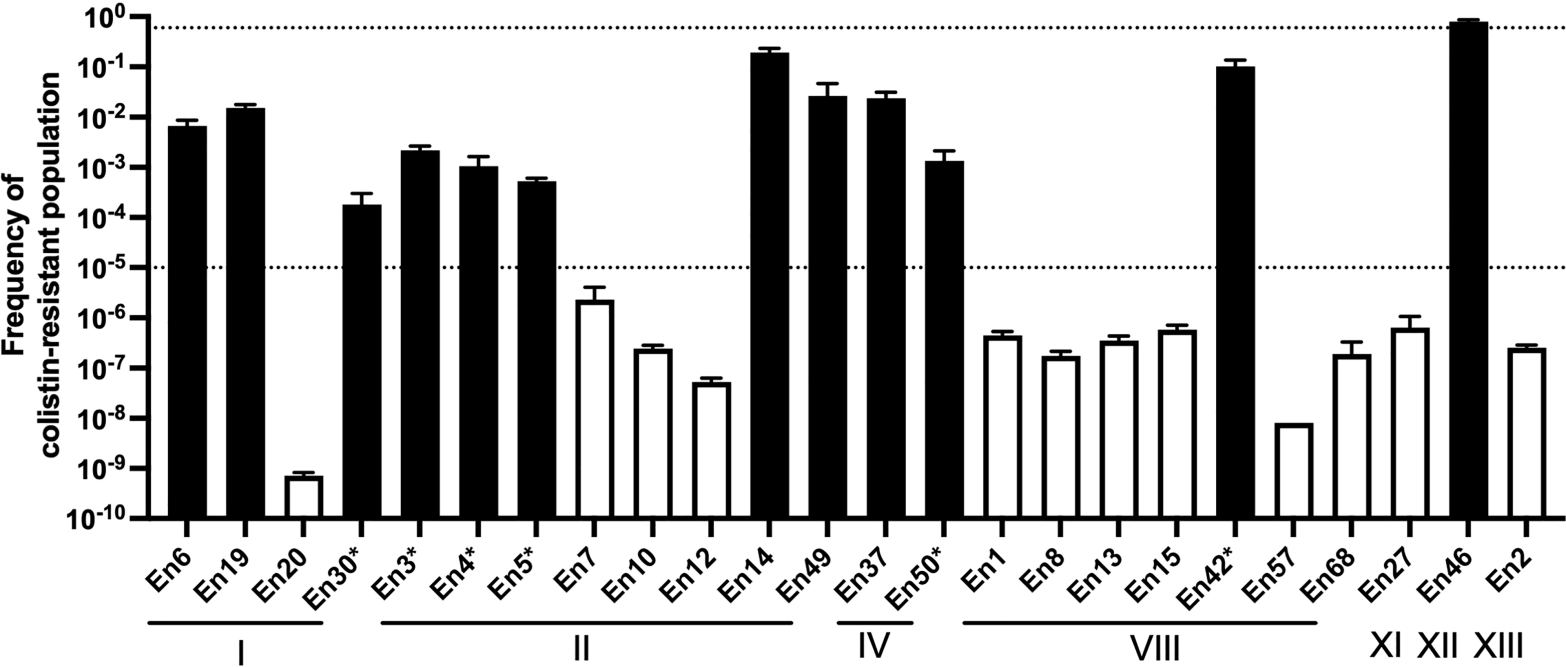
Population analysis profiling of colistin-resistant *E. cloacae* complex isolate-derived companion animals Broken lines indicate the frequency of colistin-resistant subpopulations (1 × 10^-5^) that should influence the colistin susceptibility test (after inoculation of 5 × 10^5^ cfu/ml) as determined by CLSI guidelines (Clinical and Laboratory Standards Institute, 2020). Black and white bars indicate the frequencies of colistin-resistant populations in colistin-resistant and colistin-susceptible isolate cultures, respectively. *, isolates exhibited a “skip-well” phenomenon in A. Roman numerals indicate clusters by *hsp60* phylogeny.

To evaluate a potential colistin heteroresistance phenotype, we calculated the frequency of colistin resistance in populations of *Enterobacter* spp. isolates using PAP (**Figure 4**). All the colistin-resistant *Enterobacter* spp. isolates exhibited a colistin-resistant population with a greater than 1 × 10^-5^ frequency. The frequency of the colistin-resistant subpopulation of 11 colistin-resistant *Enterobacter* spp. isolates ranged between 1.8 × 10^-4^ and 2.6 × 10^-2^. The frequency of the colistin-resistant population in the stably resistant strain En46 was 8.0 × 10^-1^ ± 2.0 × 10^-2^. Conversely, the frequency of colistin-resistant populations in colistin-susceptible isolates was lower than 1 × 10^-5^.

## Discussion

In this study, we investigated the colistin susceptibility of *Enterobacterales* derived from companion animals. To the best of our knowledge, this is the first report to evaluate the colistin susceptibility of *Enterobacter* spp. and reveal a high frequency of colistin-resistant *E. cloacae* complex isolates derived from companion animals, although colistin is not used in companion animal medicine in Japan.

The *E. cloacae* complex comprises major nosocomial pathogens, which cause opportunistic urinary, respiratory, and bloodstream infections in human (Davin-Regli et al., 2019) as well as in veterinary clinical settings (Harada et al., 2017). The *E. cloacae* complex is also clinically important as one of “ESKAPE” (Rice, 2008) pathogens due to its high frequency and multidrug resistance. These observations warrant careful monitoring of the *E. cloacae* complex as acquisition of colistin resistance may leave very few therapeutic options in the clinic. No colistin-resistant *E. cloacae* complex isolates characterized in our study were resistant to carbapenems (Harada et al., 2017). However, some colistin-resistant isolates were resistant to third-generation cephalosporins (cefotaxime and ceftazidime) due to the harboring of the genes encoding ESBL (*bla*_SHV-12_) and/or AmpC (*bla*_ACT-9_ and *bla*_ACT-10_) β-lactamases, in addition to co-resistance to fluoroquinolone and aminoglycoside. Although it is difficult to discuss that this prevalence was high or not because antimicrobial susceptibility and the antimicrobial resistance genes have not been well elucidated by using a sufficient number of *Enterobacter* spp. isolates from companion animals, these colistin-resistant isolates (*E. kobei* En3, En4, En5, and En14) may pose a significant risk due to a lack of effective treatment options.

Colistin-resistant *E. cloacae* complex isolates derived from human clinical specimens exhibit heteroresistance (Guerin et al., 2016). Colistin-heteroresistant isolates harbor the colistin-resistant subpopulation at a certain frequency (frequency of between 1 × 10^-5^ and 1 × 10^-1^) (Andersson et al., 2019), and a skip-well phenomenon is typically observed in some cases of colistin-heteroresistant isolates derived from human clinical settings (Guerin et al., 2016; Kang et al., 2019). Consistent with this, all colistin-resistant *E. cloacae* complex isolates, except one strain (En46; frequency of colistin-resistant subpopulation was 8.0 × 10^-1^), harbored colistin-resistant subpopulations at a frequency of between 1 × 10^-5^ and 1 × 10^-1^ by PAP, and some colistin-resistant isolates exhibited the “skip-well” phenomenon in the microbroth dilution method. These suggest that like the human clinical context, *E. cloacae* complex isolates from companion animals also exhibit colistin heteroresistance. Most isolates that exhibited the skip-well phenomenon displayed a colistin-resistant subpopulation at a frequency of between 10^-3^ and 10^-4^. This frequency was approximately 10-fold lower than other colistin-heteroresistant isolates that grew at all tested colistin concentrations, suggesting that the skip-well phenomenon reflects the density of colistin-resistant populations.

Previous studies demonstrated that infection of mice with colistin-heteroresistant *E. cloacae* led to colistin treatment failure (Band et al., 2016). Thus, transmission of colistin-heteroresistant *E. cloacae* complex isolates from companion animals to humans poses a potential risk to human health. Previous studies demonstrated that colistin-heteroresistant *E. cloacae* complex isolates derived from human clinical settings belonged to distinct clusters (I, II, IV, IX, X, XI, and XII) based on *hsp60* phylogeny (Guerin et al., 2016). In this study, four (I, II, IV, and XII) of the five identified clusters of colistin-resistant isolates were identical to those of human origin. In addition, the sequence types, *E. kobei* ST591 (En3, En4, and En5; *hsp60* phylogeny cluster II) (Zhou et al., 2018) and *E. asburiae* ST562 (En19; *hsp60* phylogeny cluster I) (Wang et al., 2017), identified in this study have been reported in the human clinical setting. Carriage of resistance to multiple classes of antimicrobial agents in *E. kobei* ST591 isolates (En3, En4 and En5) suggests the risk of transmission to humans where there may be few treatment options. Therefore, careful monitoring of transmission of colistin-resistant *E. cloacae* complex isolates between companion animals and humas is required in line with the One Health approach.

Previous studies have reported that the mechanisms underpinning colistin heteroresistance in *E. cloacae* complex strains are encoded on the chromosome (Kang et al., 2019). The two-component system PhoPQ causes ArnT to modify lipid A of the bacterial lipopolysaccharide with a 4-amino-4-deoxy-L-arabinose, resulting in colistin heteroresistance (Kang et al., 2019). However, the mechanism underpinning colistin heteroresistance in strains isolated from companion animals remains unclear and requires further investigation. Furthermore, the influence of *mcr* on colistin heteroresistance in *E. cloacae* requires further study, as one *E. asburiae* (En30) and one *E. roggenkampii* (En37) isolate possessed *mcr-9* and *mcr-10*, respectively (Sato et al., 2021a; Sato et al., 2021b) but had no significant colistin-resistant subpopulation frequency compared with other non-*mcr*-possessing isolates from the same *hsp60* phylogenetic cluster.

In conclusion, this study is the first to demonstrate the high-frequency isolation of colistin-resistant *E. cloacae* complex strains from companion animals in Japan. This finding provides an enhanced understanding of the spread of colistin-resistant bacteria in companion animals and highlights a strategy to prevent the spread of resistant isolates in small animal clinics and domestically. Clustering of human and companion animal-associated colistin-resistant *E. cloacae* complex isolates with resistance to multiple classes of antimicrobial agents by *hsp60* phylogeny and STs suggests the possibility of a frequent transmission between humans and companion animals. These data highlight the need for a One Health approach in tackling the spread of drug-resistant pathogens.

## Data Availability Statement

The data presented in the study are deposited in the DDBJ/ENA/GenBank repository, accession number DRX366470 - DRX366481.

## Ethics Statement

The animal study was reviewed and approved by Guidelines for Proper Conduct of Animal Experiments of the Science Council of Japan. Written informed consent was obtained from the owners for the participation of their animals in this study.

## Author Contributions

TS is responsible for the study design. KH and MU designed this study. KH assisted with sample collection. MU assisted in data analysis. SY and MH completed the written report. All authors contributed to the article and approved the submitted versions.

## Funding

This work was supported by grants from the Japan Agency for Medical Research and Development (AMED) (JP20ak0101118h0002). This work was also partly supported by a grant from JSPS KAKENHI (JP21H03622).

## Conflict of Interest

The authors declare that the research was conducted in the absence of any commercial or financial relationships that could be construed as a potential conflict of interest.

## Publisher’s Note

All claims expressed in this article are solely those of the authors and do not necessarily represent those of their affiliated organizations, or those of the publisher, the editors and the reviewers. Any product that may be evaluated in this article, or claim that may be made by its manufacturer, is not guaranteed or endorsed by the publisher.
